# Supplementary material for: Gap state analysis in electric-field-induced band gap for bilayer graphene
Source: Sci Rep. 2015 Oct 29;5:15789. doi: 10.1038/srep15789 (PMC4625181; doi:10.1038/srep15789)
Supplement: Supplementary Information [file srep15789-s1.pdf]

Supplemental information

Gap states analysis in electrostatically-opened band gap for bilayer graphene

Kaoru Kanayama[1], and Kosuke Nagashio[1,2*]
[1]Department of Materials Engineering, The University of Tokyo, Tokyo 113-8656, Japan
[2]PRESTO, Japan Science and Technology Agency (JST), Tokyo 113-8656, Japan
[*]nagashio@material.t.u-tokyo.ac.jp

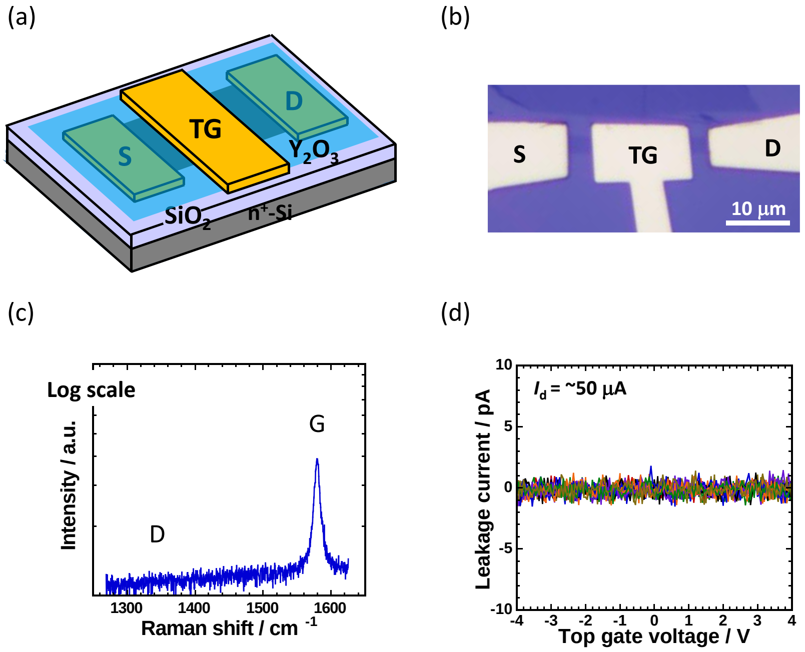

Figure S1 (a) Schematic of a dual gate graphene bilayer FET. (b) Optical micrograph of the device. (c) Raman data obtained through Y_2O_3 top gate insulator after the high-pressure O_2 annealing. (d) Top gate leakage current obtained during I_D-V_{TG} measurement. The drain current is roughly ~50 μA, while the top gate leakage current is a few pA for all different V_{BG}.

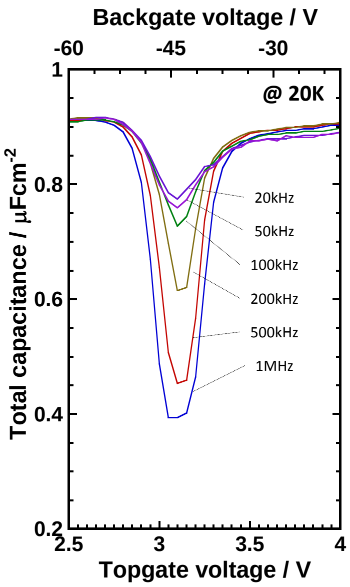

Figure S2 Frequency dependence of C_{Total} at $\overline{\mathrm{D}}$=-2.5 V/nm, where V_{TG} and V_{BG} are changed at the same time, as shown in the upper and lower transverse axes. The source-drain-topgate device without bilayer graphene channel was fabricated just near the bilayer graphene FET device on the same wafer. The capacitance of the device without bilayer graphene shows some frequency dependence. This parasitic capacitance has been removed from this data.

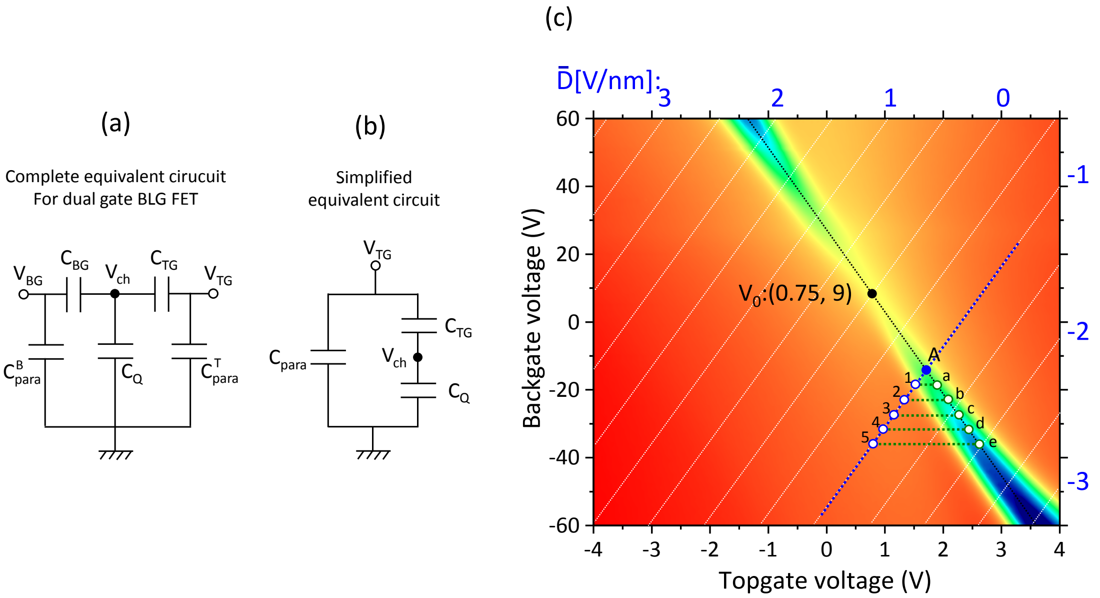

Figure S3 In case of bilayer graphene, when V_{TG} is applied under the constant V_{BG} to change E_F, the band structure is also changed at the same time. Therefore, C_Q should be extracted as a function of E_F along the constant $\bar{D}$ lines to estimate E_G. The complete equivalent circuit for the dual gate bilayer graphene FET is shown in **Fig. S3a**. In this case, the determination of V_{ch}, i.e. E_F (= eV_{ch}), is quite complicated. The equivalent circuit can be converted to **Fig. S3b**, where the contribution of C_{BG} in C_{Total} is involved through C_Q and V_{ch}. Now, V_{ch} can be calculated under the constant V_{BG} by the following equation,

$$V_{ch} = V'_{TG} - \int_0^{V'_{TG}} C'_{Total}/C_{Y2O3}\, dV'_{TG}. \qquad [A]$$

For the estimation of V_{ch} for the position “1” along $\bar{D}$ = –1 V/nm in **Fig. S3c**, C'_{Total} (differential capacitance) should be integrated from the position “a” to the position “1” along the constant V_{BG} line. This is equivalent with the integration of C'_{Total} from the position “A” to the position “1” along the constant $\bar{D}$, because the final position “1” is the same. Altough the contribution of C_{BG} is not explicitly apparent in the equation [A], the change in the band strcuture and E_F by C_{BG} are implicitly included in C'_{Total}. To obtain C_Q as a function of E_F, this calculation was iterated “1”, “2”, “3”, “4”, and “5” along the constant $\bar{D}$.

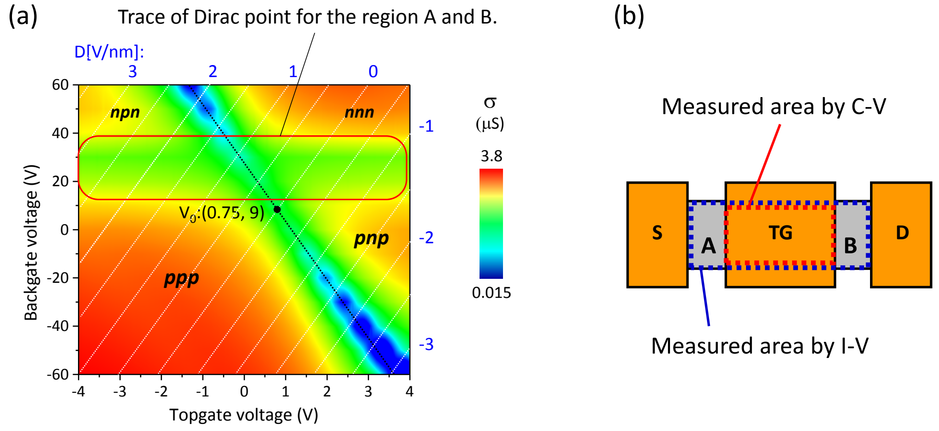

Figure S4 (a) Counter plot of σ, showing the transverse constant σ region. The access region between source(drain) and topgate (A and B in **Fig. S4b**) is only modulated by the backgate. The transverse constant σ region is formed due to the trace of Dirac point for A and B regions. Therefore, the mobility at the hatched region ($0 < \overline{D} < 1.2$) in **Fig. 3b** is relatively low.

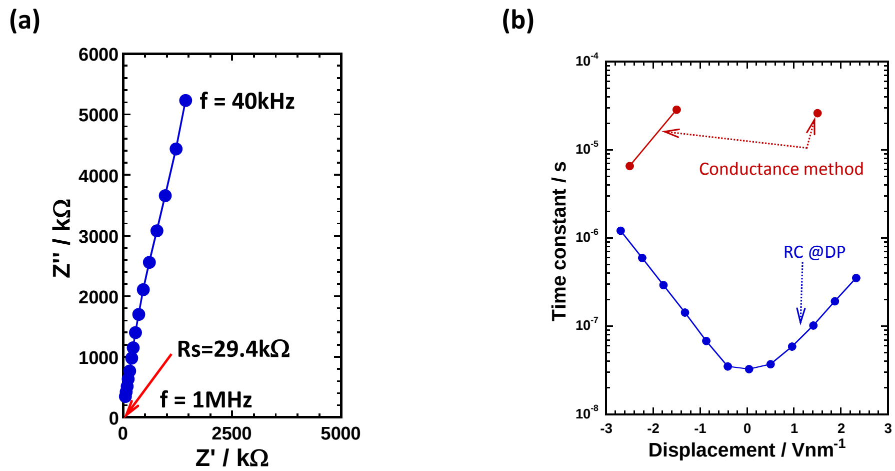

Figure S5 (a) Z'' as a function of Z'. R_s is estimated to be 29.4 kΩ. (b) Time constant as a function of $\overline{D}$ for τ_{it} and τ_{ch}, where $\tau_{ch} = R_{DP}C_{DP}$, where R_{DP} and C_{DP} are the measured resistance and capacitance at the Dirac point.
